# High-Density Lipoprotein Subfractions: Much Ado about Nothing or Clinically Important?

**DOI:** 10.3390/biomedicines9070836

**Published:** 2021-07-18

**Authors:** Knut Tore Lappegård, Christian Abendstein Kjellmo, Anders Hovland

**Affiliations:** 1 Department of Medicine, Nordland Hospital, 8092 Bodø, Norway; anders.w.hovland@gmail.com; 2 Department of Clinical Medicine, UiT The Arctic University of Norway, 9037 Tromsø, Norway; 3 Department of Gastroenterology, St. Olavs University Hospital, 7030 Trondheim, Norway; kjellmo@gmail.com

**Keywords:** high density lipoproteins, HDL, HDL-C, subclasses, subfractions

## Abstract

High-density lipoproteins (HDL) are a heterogenous group of plasma molecules with a large variety in composition. There is a wide specter in lipid content and the number of different proteins that has been associated with HDL is approaching 100. Given this heterogeneity and the fact that the total amount of HDL is inversely related to the risk of coronary heart disease (CHD), there has been increasing interest in the function of specific HDL subgroups and in what way measuring and quantifying these subgroups could be of clinical importance in determining individual CHD risk. If certain subgroups appear to be more protective than others, it may also in the future be possible to pharmacologically increase beneficial and decrease harmful subgroups in order to reduce CHD risk. In this review we give a short historical perspective, summarize some of the recent clinical findings regarding HDL subclassifications and discuss why such classification may or may not be of clinical relevance.

## 1. Introduction

An association between levels of HDL-cholesterol (HDL-C) and the risk of CHD was shown back in the 1970s, based on data both from Framingham [1] and other trials [2,3]. The findings have later been thoroughly confirmed [4]; however, it must be emphasized that although there is an association no causal relationship has been proven. Laboratory and animal experiments later showed positive effects of increasing HDL, for instance a regression of atherosclerotic plaques in rabbits fed an HDL-rich diet [5]. This in turn led to clinical trials in humans, but so far the results have been disappointing, with several agents that significantly increase HDL-C failing to reduce hard cardiovascular endpoints in rigorous trials.

As a consequence, the focus has shifted from HDL-C quantity to the cardioprotective functions of the HDL particles. The cholesterol efflux capacity (CEC)—the removal of cholesterol from foam cell macrophages in the tissues—is considered the most important function through which HDL exerts its protective effect against coronary heart disease.

Different enzymes participate in the efflux process, but it seems that the transfer via the protein ATP binding cassette transporter A1 (ABCA1) to the smaller, dense HDL subfractions HDL3b and HDL3c plays a major role [6]. The fact that HDL functionality could vary across different HDL subpopulations has led to an increased interest in subfractionating and subclassing of HDL particles. Given the number of different proteins associated with HDL, it is also highly likely that other mechanisms, such as anti-oxidative capacity and anti-inflammatory properties, contribute to the cardioprotective functions of HDL as well.

Agents that increase the level of HDL-C (primarily drugs that inhibit cholesteryl ester transfer protein (CETP)) have not been able to show clinical benefit in large cardiovascular trials, and some even showed increased morbidity or mortality [7,8,9].

It should also be noted that extremely high levels of HDL are associated with a paradoxical increase in all-cause mortality, further complicating the picture [10]. The reason for this U-shaped curve is unknown, although theories have been suggested [11].

Thus, recent guidelines regarding management of dyslipidemias and coronary heart disease focus mainly on low density lipoproteins (LDL) and do not contain any recommendations for elevating HDL-C levels [12]. However, both the European Score risk calculator and the Framingham risk calculator (USA) include HDL-C levels when assessing the risk of CHD and show that increasing levels of HDL reduce the risk of future CHD.

### 1.1. Structure of HDL

The HDL particles consist of a lipid core and a protein surface. The lipid core is a mix of a variety of lipids, including cholesterol (both esterified and non-esterified), triglycerides, phospholipids, and sphingolipids as well as small amounts of free fatty acids. The most abundant protein is Apolipoprotein A-I (Apo A-I) which is probably present in all HDL particles and accounts for around 70% of total HDL protein. It is estimated that on average there are 3–4 copies of Apo A-1 per HDL particle [13]. In addition to Apo A-I and other apolipoproteins the protein content may include enzymes (such as paraoxonase 1 (PON1), lechitin/cholesterol acyltransferase (LCAT) and the platelet inhibitor platelet-activating factor acetyl hydrolase (PAF-AH)), acute phase proteins (e.g., LPS-binding protein, serum-amyloid A), complement components C3 and clusterin (also known as ApoJ), antioxidants, transfer-proteins such as CETP, vitamin- and iron-binding proteins, serine protease inhibitors (serpins) and more. Almost 100 different proteins have been associated with HDL particles. This large number, in addition to the observation that most of these proteins are present in <1 copy per HDL particle and the fact that the proteins are not evenly distributed between the various subfractions, complicates interpretation of clinical studies. Furthermore, the proteins of HDL may also be present in different isoforms with different biological properties. As an example, it has been shown that total HDL concentration does not always correlate with efflux capacity, indicating that the responsible enzymes (e.g., PON1) may not be evenly distributed between all HDL subfractions [14].

However, it is not only the proteome that is responsible for the biological effects of HDL. The lipidome as well, in particular the negatively charged phospholipids, is of importance for the various effects of HDL, such as the anti-inflammatory, anti-oxidative, anti-apoptotic or efflux-promoting properties [15].

**Figure 1:**
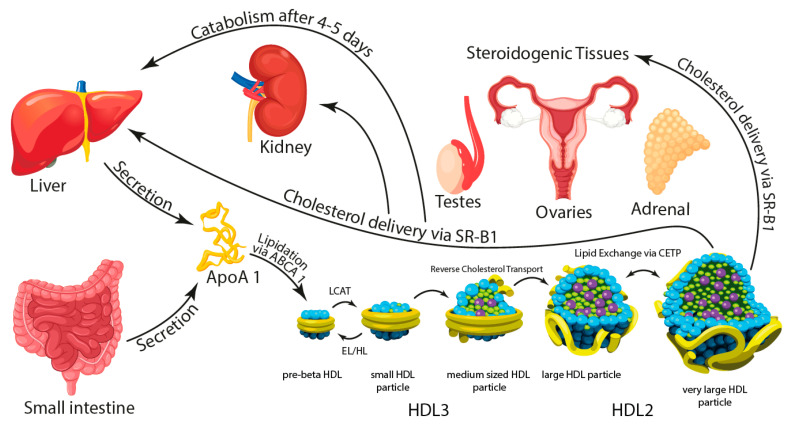
Apolipoprotein A-1 (ApoA-1) is synthesized in the liver and the intestine and secreted in a lipid-free state. Assembly of the HDL particle begins with the lipidation of the ApoA-1 protein via ABCA-1 cholesterol efflux, creating a discoidal shape termed pre-β HDL. Lipidation of pre-β HDL via LCAT results in the formation of small, spherical HDL particles. Further re-modelling of the HDL particle is a complex metabolic process that involves several enzymes. The HDL particles may grow in size by accumulating cholesterol and other lipids from peripheral tissue, a process termed reverse cholesterol transport (RCT), widely considered to be the main function of HDL. HDLs may shrink in size by delivery of cholesterol and lipids to steroidogenic tissue via Scavenger Receptor-B1 (SR-B1), peripheral tissue and the liver via endothelial (EL) and hepatic lipases (HL), or lipid exchange via CETP to apoB-containing lipoproteins. After a 4-to-5-day life cycle HDLs are permanently catabolized in the liver or kidneys.

The majority of HDL is synthesized in the liver, but other organs, in particular the intestine, contribute to a certain extent. HDL from different origins may differ in form as liver-derived HDL has been said to be more discoid compared to the spheric HDL particles from the intestine. A simplified illustration of origin and metabolism of the HDL particle is shown in Figure 1.

As will be discussed below, a number of methods are used to separate and subclassify HDL. The variety in protein as well as lipid content opens the possibility for even more ways to do this, not based on density, size or shape, but for instance based on function.

### 1.2. Methods of Separation

As previously mentioned, HDL is an extremely heterogenous class of lipoproteins and is regarded and discussed as a group merely on the basis of the hydrated density of its particles.

This density is between 1063 mg/mL and 1210 mg/mL. Through ultracentrifugation HDL has traditionally been divided into two groups: the more lipid rich HDL2 particles with a density of 1063–1125 mg/mL and the more protein rich HDL3 particles with a density of 1125–1210 mg/mL. But even after such ultracentrifugation a considerable heterogeneity persists, with large differences in size, shape and electric charge, as well as lipid and protein composition. Approximately 60% of total HDL is in the HDL3 subclass, and these particles are in general smaller and contain less cholesterol than the larger, cholesterol-rich HDL2 which accounts for 40% of total HDL.

Further differentiation can be made based on size, the most commonly used method being non-denaturing polyacrylamide gel electrophoresis which divides the two fractions into five: HDL2b (9.7–12 nm), HDL2a (8.8–9.7 nm), HDL3a (8.2–8.8 nm), HDL3b (7.8–8.2 nm) and HDL3c (7.2–7.8 nm). Another method is nuclear magnetic resonance, which divides HDL into large (8.8–13 nm), medium (8.2–8.8 nm) and small (7.3–8.2 nm) particles.

Additional methods for separation of HDL are available and used with different frequency in studies of HDL. They are based on different physico-chemical properties of the HDL molecules and separate the particles on the basis of size, charge, density etc. Not unexpectedly, it has been shown that different separation methods may provide different estimates of HDL particle numbers so that results are not immediately comparable [16]. Furthermore, cost, size, availability, operator friendliness and a number of other factors imply that some methods are relevant for clinical purposes, whereas others probably will be restricted to research. Matera and coworkers performed a systematic comparison of five methods: ultracentrifugation/vertical auto profile, NMR, two-dimensional gel electrophoresis, ion mobility and enzyme-linked immunosorbent assay based on Apo A-1 [17]. Hafiane et al. published a review of several different methods for HDL subfractioning [18], and newer methods have also been developed and suggested [19]. In 2009 the American Heart Association (AHA) published a framework for evaluating novel risk markers [20]: a new risk marker should predict future outcomes in prospective studies, add predictive information to established risk markers, improve clinical outcomes and be determined as cost-effective when compared to established risk markers. This framework sets a very high bar for novel risk markers and should be considered a guideline, but a minimum requirement is documentation that the risk marker adds predictive information when compared to the established risk markers. Most of the published prospective and clinical trials evaluating the use of HDL subfractions to predict outcomes have used one of the patented laboratory tests or patented in-house systems that are available to clinicians: Lipoprint HDL^®^ (gel electrophoresis), Cardio IQ^®^ (ion mobility), NMR LipoProfile^®^ (NMR) and, until recently, Vertical auto profile (VAP)^®^ (ultracentrifugation) (Table 1).

All these methods have their advantages and disadvantages, but the challenge arises when trying to compare results from different trials that have used different methods. The “same” HDL subfractions obtained by different methods may contain HDL particles with very different properties, making it impossible to translate findings from one study to another if different separation techniques are used. A consensus nomenclature was suggested around ten years ago [21], but the current literature still includes a number of ways to describe the different subgroups because no gold standard or reference method exists.

Another challenge with the different methods of separation is the fact that the process itself may affect the HDL particles to varying degrees. For instance, the high ionic strength associated with density gradient ultracentrifugation may cause changes in surface protein structure [22,23]. An important function of HDL is the transport of cholesterol from peripheral cells to the liver. The capacity of HDL to accept cholesterol released from cultured cells is the principle of the cholesterol efflux assay, but measuring efflux capacity in a subfraction obtained by one separation method may be different from another as the separation process (as mentioned above) or the assay itself may have had an impact. The fact that HDL function such as CEC can be determined by different assays [24,25] further complicates the picture. Finally, frozen storage can also affect both the structure and the function of HDL particles [26]. Thus, comparing results from studies that have used different separation methods should be done with caution.

## 2. Subfractions and Association with Clinical Disease

A number of epidemiologic studies have found associations between particle size or subfractions of HDL on one side and various clinical conditions on the other. Whether such correlations are a result or a cause remains to be determined. Early epidemiological studies indicated that low levels of HDL2 were a better predictor of CHD than total HDL or HDL3 [27,28]. Such a correlation could be explained by the fact that low HDL2 is associated with a number of the components of the metabolic syndrome, such as increased weight, insulin resistance, reduced glucose tolerance and increased fasting blood sugar [29]. This is in line with recent findings that obese individuals have increased levels of the smaller HDL3 and lower levels of HDL2 compared to individuals who were of normal weight or overweight (but not obese) [30]. Montero et al. recently observed that a weight reduction program increased the large HDL-C subfraction in a group of obese adolescents [31].

However, findings that HDL2 is the best predictor of CHD are conflicting as other trials and meta-analyses show that HDL3 is superior [32,33]. Although such results seem mutually exclusive, they may merely be a result of different populations being studied. Such differences may be the inherent risk of CHD in the population, genetic predisposition, whether the subjects used statins and a number of other factors. Gender may be one such factor, as it has been shown that HDL composition differs between males and females, both in atherogenic and non-atherogenic phenotypes with males in general having a more atherogenic HDL composition than females [34]. Another factor may be the level of LDL as this affects HDL cholesterol efflux capacity, at least in individuals with familial hypercholesterolemia [35].

The associations to various specific heart diseases have frequently been studied, but a number of other conditions have also been subject to investigation. For instance, in patients with Alzheimer’s disease it was found that the small subclass of HDL was reduced, and this correlated with cognitive performance [36], in contrast to a study of patients with mild cognitive impairment where the levels of small-sized HDL particles were increased compared to controls [37]. In this latter trial, patients with Alzheimer´s disease did not differ from healthy controls. In both these trials the investigators used Lipoprint^®^, a separation method based on electrophoresis (see above), so in this comparison the difference in results cannot be explained by different separation methods. Thus, the possible link between cholesterol and Alzheimer´s disease is still elusive.

Dividing HDL into groups HDL2 and HDL3, Degoricija and coworkers found that lower levels of HDL3 at admission for acute heart failure were associated with increased mortality after 3 months, whereas levels of HDL2 or total HDL showed no such correlation [38]. In patients with chronic heart failure followed for a median of 4 years, it was shown that a higher mean HDL particle size correlated with increased mortality. Particle size was determined using NMR spectroscopy [39]. This is in line with the findings of Hunter and coworkers, who demonstrated a correlation between particle size and left ventricular ejection fraction. Patients with heart failure with reduced ejection fraction (HFrEF) had a larger mean HDL particle size compared to patients with heart failure with preserved ejection fraction (HFpEF), who in turn had larger particles than healthy controls [40]. Again, NMR was used to determine size and subfractions.

The nature of the correlation between heart failure and HDL is unclear, but there might be a link through the gut microbiome [41]. It is well known that the microbiome is altered in heart failure [42,43,44], and it has also been shown through NMR that there is an association between certain microbial families and genera and levels of LDL- and HDL- subfractions, e.g., the genera LachnospiraceaeNC2004group and LachnospiraceaeFCS020group [45].

However, although there might be a link between particle size and heart failure, Generoso and coworkers found no definite association between total HDL or levels of HDL2 and HDL3 (as determined by ultracentrifugation, VAP) and coronary calcium score in healthy individuals [46]. This is in line with another trial where Kidawa and coworkers found no differences in HDL subgroups between patients admitted for ST elevation myocardial infarction, non-ST elevation myocardial infarction and unstable angina pectoris. They used gel electrophoresis (Lipoprint^®^) to quantify the various fractions [47]. In a study of 170 individuals with increased cardiovascular risk, Aneni and coworkers found no definite association between HDL subgroups (assessed by ion mobility) and coronary calcification, although there was some correlation for certain LDL subgroups [48]. This is in contrast to the findings of Chaudhary and coworkers, who found that low levels of HDL3 (and total HDL) correlated with the severity of coronary artery disease in 300 patients undergoing coronary catheterization [49]. Furthermore, it has also been shown that low levels of HDL3 increases the risk of periprocedural myocardial injury in a setting of elective percutaneous coronary intervention in patients with stable angina pectoris [50]. Low levels of HDL3 have also been associated with increased arterial stiffness in a population-based prospective study of >1400 individuals, but there was no association with HDL2 or total HDL [51]. Interestingly, patients with abdominal aortic aneurysms (AAA) have HDL with impaired ability to cholesterol efflux, when compared to healthy controls [52,53], possibly due to antibodies against HDL [54]. However, reduced efflux capacity does not seem to correlate to AAA progression [55]. Such a change of focus from merely measuring the level of the various subfractions to evaluating their functional capacity might be a way to explain apparent discrepancies between various previous trials. As an example, Carnuta and coworkers demonstrated that in patients with coronary artery disease (acute coronary syndrome (ACS) and stable angina pectoris (SA)), HDL was dysfunctional in terms of, e.g., anti-inflammatory potential, and that there were differences between patients with SA versus patients with ACS. When HDL from these patients was incubated with activated endothelial cells, the release of inflammatory mediators was much higher with HDL from ACS patients compared to SA patients [56]. Patients in hemodialysis are at especially high risk of cardio- and cerebrovascular events, and in a prospective study it was shown that individuals who during follow up experienced such events had higher levels of HDL3 and lower levels of HDL2b when compared to dialysis patients with no such events [57]. Gluba-Brzòzka et al. also studied patients with end-stage renal disease in hemodialysis and compared to unaffected controls the patients with renal disease had higher levels of the large HDL particles as measured by the Lipoprint^®^ system; however, no important differences were found for the LDL-subfractions [58]. This could indicate that the composition of HDL-subgroups may be of importance regarding the increased risk of atherosclerotic disease in patients with end stage renal disease.

Inflammation in general and the complement system in particular are central parts of the atherosclerotic process [59,60], and in a recent population study it was shown (using NMR) that there is a positive correlation between circulating levels of complement component C3 and the number of small HDL particles, and an inverse relationship between C3 and large HDL particles [61]. The link between inflammation and coronary heart disease was addressed in the JUPITER trial [62], where patients with normal LDL but elevated high-sensitivity C-Reactive Protein were treated with rosuvastatin. A sub-analysis demonstrated that the HDL particle number was the best predictor of disease, with an inverse association to a combination of cardio- and cerebrovascular events [63].

Investigating patients with type 1 diabetes, Vaisar and coworkers found that less vascular complications was associated with an increased level of medium-sized HDL (ion mobility) [64].

In a different study using Lipoprint^®^, the investigators found that patients with a long history of diabetes mellitus type 2 had lower levels of HDL-6 and intermediate HDL compared to newly diagnosed diabetes patients and healthy controls [65]. Lipoprint^®^ separates HDL into 10 subfractions where 1–3 corresponds to small, 4–7 corresponds to intermediate and 8–10 corresponds to large HDL particles. Other studies have demonstrated that patients with DM2 have dysfunctional HDL [66]. In patients with pulmonary arterial hypertension, it has been shown that reduced levels of the small HDL4 particle (as determined by NMR which divides HDL into four groups with HDL1 being large, HDL2 and HDL3 intermediate and HDL4 being small) was associated with a high mortality [67]. A low level of small size HDL (as determined by Lipoprint^®^) was also found to correlate to an unfavorable outcome in stroke patients [68].

To what extent differences in HDL level and composition can be attributed to genetic factors is not fully understood, but it has been shown that children with FH had HDL which differed from healthy children with respect to particle concentration as well as lipid content [69].

Thus, numerous various clinical conditions have been associated with different HDL subfractions, but a lot of research remains both to determine any causal relationship and to explain conflicting results when different methods of separation and classification have been used.

## 3. Interventions

As the total level of HDL is associated with cardiovascular morbidity and mortality, and as different subgroups may predict such morbidity differently, a lot of research has focused on drugs that can increase HDL and thus, maybe reduce cardiovascular morbidity and mortality. Fibrates and niacin are both drugs that increase HDL but have failed to produce convincing evidence regarding hard endpoints. Fibrates increase HDL through increasing hepatic Apo A1 and Apo A2 production, whereas niacin increases HDL through inhibition of hepatic microsomal diacylglycerol acyltransferase-2 and inhibition of Apo A1 uptake [70]. The effect on HDL function, rather than HDL level, may be one explanation and it has been shown that these drugs behave quite differently in this manner [71]. Similarly, the trapibs—drugs that inhibit the cholesteryl ester transfer protein (CETP)—have so far not provided the expected reduction in cardiovascular events and may even cause an increase through mechanisms currently not known [8,72]. However, it was recently shown that inhibition of CETP with anacetrapib in a mouse model of sepsis both preserved HDL levels and improved survival, possibly by reducing a proinflammatory cytokine response [73].

Nevertheless, it has been documented that there is an association between CETP and subclasses in that individuals (patients and controls) with higher CETP levels have decreased levels of HDL2a and HDL2b [74].

Moderate intake of alcohol has been shown to have a certain protective effect against coronary heart disease as compared to both high alcohol consumption as well as no consumption at all. It is possible that this effect in part is through increased HDL, and both HDL2 and HDL3 seem to be of importance [75].

Smaller trials with different types of intervention have produced conflicting results regarding effect on HDL subfractions and function. For instance, a program of structured exercise over 6 months had no effect on levels of HDL2 or HDL3 or efflux capacity in a group of patients with peripheral artery disease [76]. However, in a different study, weight loss and exercise reduced the amount of small HDL particles (measured by Lipoprint^®^) in a group of subjects with obesity [77].

In a group of morbidly obese patients undergoing bariatric surgery we found that this intervention increased total HDL but had no effect on efflux capacity [78]. These findings were in part confirmed by Coimbra et al. who, using the same separation process, found that bariatric surgery increased both total HDL as well as the number of large and intermediate particles, whereas the number of small HDL particles was reduced [79]. A relative increase in the larger, lipid-rich HDL2 subfraction following bariatric surgery has also been reported by others [80]. Bariatric surgery is known to alter the gut microbiome, and this might be one mechanism through which it affects HDL concentration and subfractions.

Levels of HDL can also be affected by poly-unsaturated fatty acids, at least in children and adolescents with depression [81]. In their study, omega-3 increased the level of large and reduced the level of small HDL particles (determined by Lipoprint^®^). Similar findings were obtained by Yang and coworkers, who investigated the effect of two different fish oils (eicosapentaenoic acid (EPA) and docosahexaenoic acid (DHA)) on a number of lipid parameters in a crossover trial in 30 healthy individuals. They found no effect on total HDL or cholesterol efflux capacity, but both fish oils increased large and decreased medium size HDL particles, as determined by NMR [82]. These results are in line with the results of a large, epidemiologic trial of >26,000 women were the investigators showed that self-reported high intake of total omega-3 fatty acids, DHA and @-linoleic acid were all associated with a larger HDL particle size [83]. Polyunsaturated fat has also been shown to improve certain functions of HDL in mice [84].

Patients with type 2 diabetes often have an unfavorable lipid profile, but administering atorvastatin to reduce LDL does not seem to affect HDL level or total efflux capacity [85]. In this study, ultracentrifugation was used to separate HDL.

The effect of proprotein convertase subtilisin/kexin type 9 (PCSK9) inhibition on HDL subfractions is unclear. Using Lipoprint^®^, we found no effect in our pilot trial with evolocumab [86], but these findings were challenged in a recent, larger trial with both alirocumab and evolocumab where such treatment increased medium sized HDL and decreased very large HDL (determined by NMR) [87]. Li and coworkers found that PCSK9 inhibition in combination with rosuvastatin after an acute coronary syndrome increased HDL more than rosuvastatin alone, and that the subfraction (as determined by NMR) HDL3+4 increased more than HDL1+2 [88].

## 4. The Clinical Challenge

Abundant evidence points to the fact that low levels of HDL are associated with increased risk of CHD, but it should be kept in mind, as mentioned earlier, that the curve is U-shaped and that very high HDL levels is associated with an increased risk. Thus, continued research is needed to determine how the “right” concentration of HDL exerts its beneficial effect, how the various subfractions contribute, what role the composition of proteins and lipids plays and how future therapies may modify the effects of HDL. However, as trials with agents known to increase HDL so far have not been able to show positive effects on hard clinical endpoints and modification of specific subfractions is not available, there is at this time, in our opinion, no strong incentive for the clinician to determine HDL subfractions. This is supported by the fact that HDL subfractioning can be both costly and time-consuming. Thus, routine determination of the various subfractions is not warranted. However, as a low level of total HDL-C definitely increases CHD risk, identifying such patients should prompt the treating physician to aggressively address other risk factors the patients may have, and where intervention has a well-documented effect, including LDL levels, smoking habits, diabetes, weight, physical inactivity etc. Several studies have shown that a large proportion, in some populations a majority, of patients in primary or secondary coronary prophylaxis do not achieve their treatment goals [89,90,91]. Increased focus on these goals will reduce residual risk, but in parallel continued research on agents increasing total HDL and in particular beneficial subfractions is warranted.

## Figures and Tables

**Table 1:** Commercially available tests that report HDL subfractions.

Separation Technique	Commercially Available As	Availability	Subfractions Reported	Subfraction Based Separation
**Gel electrophoresis (GE)** 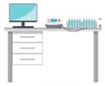	In-house system; Lipoprint HDL ©	- Small, affordable in-house system - Laborious	Particle concentrations for small, medium and large HDL	Samples are loaded into wells of an agarose or acrylamide gel and subjected to an electric field, separating particles based on size and charge
**Nuclear magnetic resonance (NMR)** 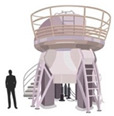	Patented laboratory test; NMR LipoProfile ©, The Lipoprotein Fractionation NMR test In-house system; Vantera ©	- Shipping and handling - Expensive tests - Expensive in-house system.	HDL particle number, large HDL particle number, HDL size	Separates lipoprotein particles from the derived amplitudes of their spectroscopically distinct lipid methyl group NMR signals
**Ion mobility (IM)** 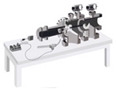	Patented laboratory test; Cardio IQ ©	- Shipping and handling, only available in the US - Expensive tests	Particle concentrations for total HDL-P, small, medium and large HDL.	Gas-phase (laminar flow) electrophoresis to separate lipoprotein particles on the basis of size
